# Microfluidic Chip for Molecular Amplification of Influenza A RNA in Human Respiratory Specimens

**DOI:** 10.1371/journal.pone.0033176

**Published:** 2012-03-22

**Authors:** Qingqing Cao, Madhumita Mahalanabis, Jessie Chang, Brendan Carey, Christopher Hsieh, Ahjegannie Stanley, Christine A. Odell, Patricia Mitchell, James Feldman, Nira R. Pollock, Catherine M. Klapperich

**Affiliations:** 1 Department of Mechanical Engineering, Boston University, Boston, Massachusetts, United States of America; 2 Department of Biomedical Engineering, Boston University, Boston, Massachusetts, United States of America; 3 Department of Pediatrics, Division of Pediatric Emergency Medicine, Boston Medical Center and Boston University School of Medicine, Boston, Massachusetts, United States of America; 4 Department of Emergency Medicine, Boston Medical Center and Boston University School of Medicine, Boston, Massachusetts, United States of America; 5 Division of Infectious Diseases, Beth Israel Deaconess Medical Center, Harvard Medical School, Boston, Massachusetts, United States of America; Northeastern University, United States of America

## Abstract

A rapid, low cost, accurate point-of-care (POC) device to detect influenza virus is needed for effective treatment and control of both seasonal and pandemic strains. We developed a single-use microfluidic chip that integrates solid phase extraction (SPE) and molecular amplification via a reverse transcription polymerase chain reaction (RT-PCR) to amplify influenza virus type A RNA. We demonstrated the ability of the chip to amplify influenza A RNA in human nasopharyngeal aspirate (NPA) and nasopharyngeal swab (NPS) specimens collected at two clinical sites from 2008–2010. The microfluidic test was dramatically more sensitive than two currently used rapid immunoassays and had high specificity that was essentially equivalent to the rapid assays and direct fluorescent antigen (DFA) testing. We report 96% *(CI 89%,99%)* sensitivity and 100% *(CI 95%,100%)* specificity compared to conventional (bench top) RT-PCR based on the testing of n = 146 specimens (positive predictive value = 100%*(CI 94%,100%)* and negative predictive value = 96%*(CI 88%,98%)*). These results compare well with DFA performed on samples taken during the same time period (98% *(CI 91%,100%)* sensitivity and 96%*(CI 86%,99%)* specificity compared to our gold standard testing). Rapid immunoassay tests on samples taken during the enrollment period were less reliable (49%*(CI 38%,61%)* sensitivity and 98%*(CI 98%,100%)* specificity). The microfluidic test extracted and amplified influenza A RNA directly from clinical specimens with viral loads down to 10^3^ copies/ml in 3 h or less. The new test represents a major improvement over viral culture in terms of turn around time, over rapid immunoassay tests in terms of sensitivity, and over bench top RT-PCR and DFA in terms of ease of use and portability.

## Introduction

In a normal year, the influenza virus infects millions of individuals [1] causing approximately 350,000 hospitalizations and 50,000 deaths in the United States [2], [3]. Furthermore, when genetic rearrangements result in antigenic shift in the virus, a pandemic strain can result [4]. In April 2009, worldwide surveillance efforts identified the emergence and rapid spread of a novel influenza A strain, which reached pandemic levels as defined by the World Health Organization (WHO) in early June of 2009 [5]. As of August 2010, worldwide more than 214 countries and overseas territories or communities had reported laboratory confirmed cases of pandemic influenza H1N1 2009, including 18,449 deaths [6].

The most recent pandemic highlighted weaknesses in the methods widely used to diagnose influenza: rapid immunoassays (antigen tests), direct fluorescent antigen testing (DFA), and viral culture methods (culture followed by immunofluorescence or hemagglutination assay for virus identification) [7]. During the pandemic, rapid influenza tests on the market were widely used and found to be dramatically lacking in sensitivity [8], [9], [10], [11] such that the Centers for Disease Control and Prevention (CDC, Atlanta, GA) recommended that a negative test result be ignored for clinical decision-making [12]. Although the DFA test worked well during the 2009 H1N1 pandemic [13], [14], the labor-intensive nature of the test and potentially subjective aspects of results interpretation somewhat reduced its utility [7]. While now being debated, viral culture remains the “gold standard” test; however, it requires 2–7 days to complete and lacks the ability to distinguish between different respiratory viruses and between influenza virus types without follow up testing [7]. Alternatively, polymerase chain reaction (PCR) testing provides a faster turnaround time, high sensitivity and high specificity. The vast amount of PCR data generated during the 2009 H1N1 pandemic supported the hypothesis that PCR performs as well or better than culture, including the ability to verify and differentiate influenza types and subtypes [8], [9], [15].

Since the 2009 H1N1 pandemic, several commercial PCR tests have been United States Food and Drug Administration (FDA) approved, including the Xpert™ Flu A panel (Cepheid, Sunnyvale, CA), the Iquum Liat™ Influenza A/2009 H1N1 test (Iquum, Marlborough, MA), the Luminex xTAG® Respiratory Viral Panel (Luminex, Austin, TX) and the Prodesse ProFAST™ assay (Gen-Probe, San Diego, CA). The Luminex and Gen-Probe tests require off-chip sample preparation either by hand or using a separate automated sample preparation system, requiring either a bench top kit and centrifuge or additional instruments and reagents. The Cepheid and Iquum tests do include sample preparation in the cartridge, but the tests are not Clinical Laboratory Improvement Amendments (CLIA) waived or point-of-care (POC). Clinical utility would be greatly enhanced by a CLIA waived molecular test that could be performed at the bedside, since a CLIA waiver allows testing and analysis of results to take place outside of the clinical laboratory by trained personnel. Here we demonstrate a proof-of-concept microfluidic chip using clinical specimens collected over a two-year period from patients with suspected influenza. The microfluidic assay uses a nucleic acid extraction method and a PCR thermal cycling strategy chosen for their relative ease and simplicity.

Since the introduction of the concept of the micro total analysis system (μ-TAS) in 1990 [16] and the introduction of the first PCR chip in 1993 [17], miniaturization of PCR devices has been extensively studied [18], [19]. Improvements have included faster amplification [20], [21], reduced sample and reagent consumption [22], [23], single-use chip materials [24] and increased integration of the PCR device with sample preparation and detection modules [25], [26], [27], [28], [29]. Only a few reports have demonstrated integrated chips with molecular diagnostic capabilities using complex clinical specimens [30], [31], [32]. Others have demonstrated on chip RT-PCR with simulated human specimens [33], [34], [35]. Here we focus on clinical specimens collected during the 2008–2010 influenza seasons. Clinical specimens present many difficulties including inhomogeneity, variations in viral load, different levels of contaminating human cells and blood, the presence of PCR inhibitors after nucleic acid extraction [36], [37] and unanticipated chemical and/or physical reactions between the specimens and the test reagents or the device itself [38]. The single-use chip proposed here incorporates nucleic acid extraction and reverse transcription-PCR (RT-PCR) in a single chip with simple thermal and fluidic control. The single use feature decreases the possibility of cross contamination between specimens, and the small form factor makes it a good candidate for true POC testing.

## Results

### Overview of Experimental Design

A total of 626 specimens were collected from two sites during 2008–2009 and 2009–2010 influenza seasons. All of the samples were characterized in our laboratory using bench top RT-PCR, which we define here as the reference method. In order to determine the analytical limit of detection for the single use influenza A assay, the microfluidic assay was first tested using a laboratory strain of influenza A (VR-1469, H1N1, A/PR/8/34 obtained from ATCC, Manassas, VA). The limit of detection determined using this laboratory strain was 10^5^ copies per milliliter of input sample (**Figure S1**). Before working with the human samples, the assay was optimized for bovine serum albumin (BSA), magnesium ion, and enzyme concentrations in order to push the limit to 10^4^ cp/ml (**Figures S2, S3, S4**). Next, nasopharyngeal aspirate (NPA) and nasopharyngeal swab (NPS) specimens that were collected with Institutional Review Board approval from patients presenting with symptoms consistent with influenza to two Boston hospitals, as described in the methods, were tested by bench top RT-PCR. A total of 146 samples were then selected (73 positive and 73 negative by bench top RT-PCR) from this pool, without regard to viral load, for testing with the microfluidic assay. Results of DFA (n = 106) and rapid immunoassay (n = 119) testing that had been performed on specimens as part of routine care at each of the two hospitals were also compared to results obtained from testing of those specimens by our bench top PCR reference standard. While there was some overlap in the sample sets, in general, different samples were used to compare each method (microfluidic, DFA, and rapid immunoassay) to bench top RT-PCR. Reagents described in the CDC research-use only (RUO) protocol for real-time PCR of influenza A and B were used in the gold-standard bench top PCR assay [39].

### Microfluidic assay: human specimens

A set of human nasopharyngeal swab (n = 35) and aspirate (n = 111) samples were tested in the single use microfluidic assay (n = 146). All of the samples (n = 146) were also tested for both influenza A and influenza B in our laboratory using bench top quantitative RT-PCR. Quantification of the microfluidic assay output is summarized in **Table S1**. The specimens tested in the microfluidic assay included n = 73 specimens positive for influenza A and n = 73 specimens negative for influenza A by bench top RT-PCR. The 73 influenza A negative specimens included 20 samples that tested negative for influenza A but positive for influenza B by the bench top reference assay in our laboratory.

**Figure 1:**
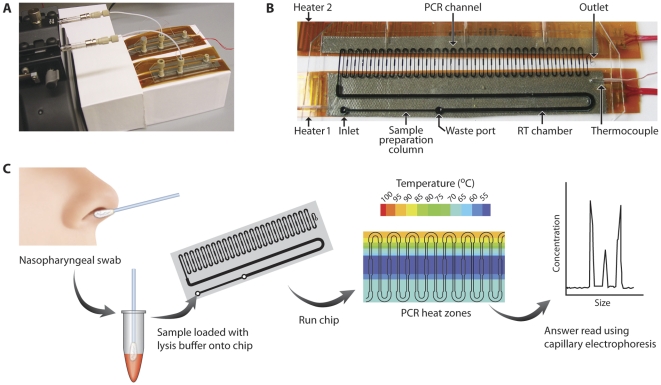
The microfluidic assay flow. (A) Image of two microfluidic chips with attached thin film heaters and the two-barrel syringe pump. Glass syringes were connected to each chip with flexible tubing to load reagents and samples. Three ports were glued at the inlets of SPE channel and the waste port, and the outlet of the PCR channel. (B) Channel design with three sections: sample preparation (SPE), RT chamber and continuous flow PCR channel. Two fixed resistance heaters are attached via thermal tape to the bottom of the chip. Fluid flow between the channels was linear, and changes in fluid resistance allowed for valveless operation. The depth is 500 µm for SPE and RT channels, and the PCR channel is 100 µm deep. The widths are 500 µm for the SPE column, 1 mm for the RT chamber, and vary from 200 to 400 µm for the wide and narrow sections of the continuous flow PCR channel. The chip is 70 mm in length, 25 mm in width and 1.4 mm in height. (C) Microfluidic assay process flow. The nasopharyngeal sample is mixed with lysis buffer, applied to the chip, the chip is run, and the PCR products are read using a commercial capillary electrophoresis chip.

The disposable microfluidic assay included sample injection after mixing with lysis buffer (see Methods), nucleic acid isolation by solid phase extraction (SPE) (nucleic acid extraction), reverse transcription (RT) and continuous flow polymerase chain reaction (PCR) (**Figure 1**). Products were detected off-chip by capillary electrophoresis. Each test was completed in 3 hours or less. The test time included approximately 110 min for sample preparation, 30 min for RT, 15 min for polymerase activation, 20 min for PCR, and 5 min for off-chip detection. It took more than 4 hours to complete the same steps for the gold standard RT-PCR assays. The sample preparation is the rate-limiting step in the current chip design. Simple modifications to greatly reduce the time spent on this step are covered in the discussion. The bench top assays also required many more handling steps. The PCR products were verified by gel electrophoresis (2100 Bioanalyzer and 12% PAGE) and a subset of the positive results were verified by sequencing. The on-chip PCR product yield ranged from 43 pg/µl to 1.5 ng/µl. The majority (77%) of the amplicons had endpoint concentrations between 0.1–1.0 ng/µl. Periodically, products from the positive reactions (specifically, every 10^th^ product) were sequenced for quality control. The sequences were submitted to the GenBank database (accession numbers HM370561-HM370565; HQ902243-HQ902250). A BLAST search of the sequenced region returned results of 98–100% homology for all sequences (n = 13) with the influenza A M1 gene in segment 7 from GenBank. Next, all of the chip product sequences were aligned with the standard reference influenza strain A/PR/8/34 M1 gene (position number 171 to 276 in M1, accession number NC_002016).

**Figure 2:**
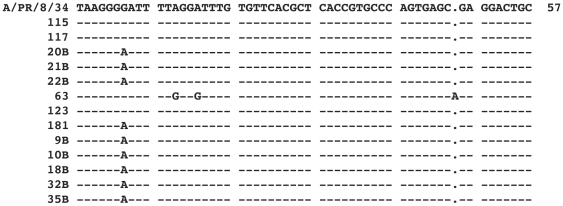
Sequence alignment of microfluidic assay RT-PCR products to standard reference influenza strain A/PR/8/34 M1 gene (position number: 193–252). The sequence presented here excludes primer sequences.

**Figure 3:**
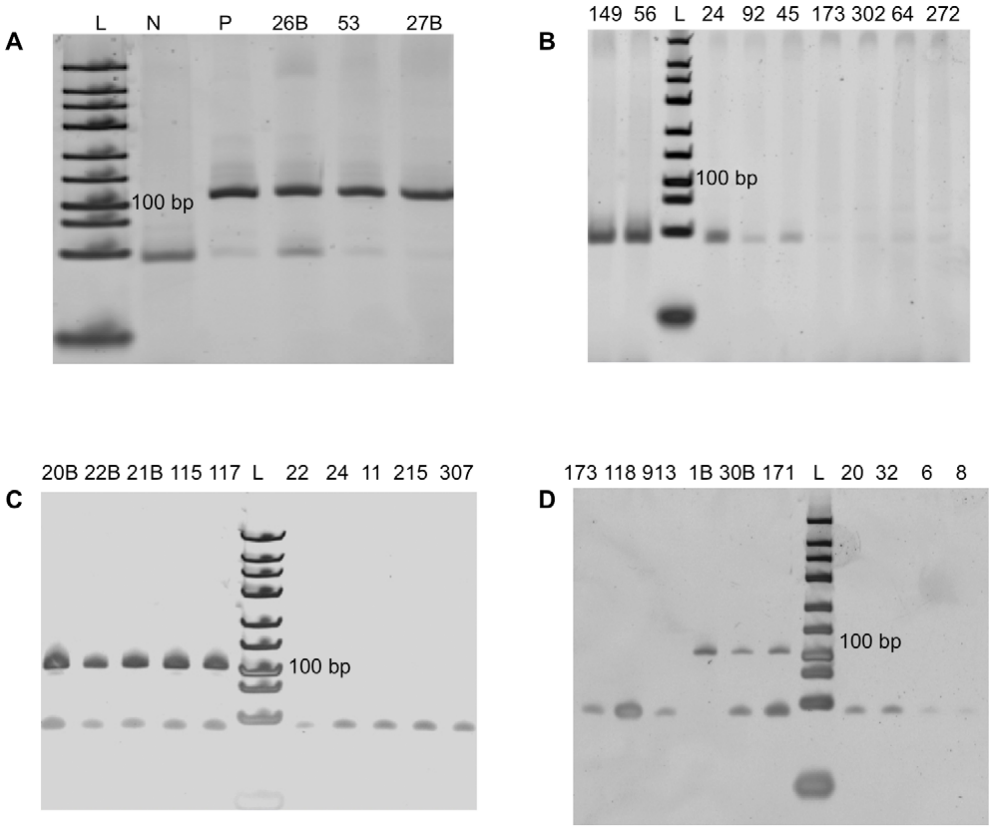
Representative on-chip RT-PCR products on 12% polyacrylamide gel. P: cultured influenza A virus (A/PR/8/34) microfluidic assay result. N: negative control (nuclease free water) in microfluidic chip result. Positive specimens: 26B, 53, 27B, 20B, 22B, 21B, 115, 117, 31B, 30B, 171. negative specimens: 149, 56, 24, 92, 45, 173, 302, 64, 272, 22, 24, 112, 150, 307, 173, 118, 91, 20, 32, 6, 8. L: GeneRuler™ Ultra Low Range DNA Ladder (Fermentas, Glen Burnie, Maryland).

All sequences were confirmed to be from influenza A; 12/13 of the specimens sequenced had greater than 98.2% homology with the reference, and one was 94.7% homologous. We observed variance of the sequence at position 199 in 9/13 sequences between guanine and adenine, and further sequence changes were observed in specimen ID 63 (**Figure 2**). To determine if these positions were variable in M1 and to determine the frequency of variations at these positions, we retrieved all (n = 739) GenBank M1 sequences isolated from humans during the same time span of our study, 2008–2010, in North America from the NCBI's Influenza Virus Resource database [40]. The sequences were aligned using the Influenza Virus Resource alignment tool. Adenine was found to be a common base at position 199, occurring in 66% of the sequences. Three positions in specimen ID 63 were altered; the same 2/3 variations were observed at low frequency in the database (0.3%–0.7%)), however we did not find the third variation, an insertion, in the database. All positive reactions and every 10^th^ negative reaction (a subset is shown in **Figure 3**) were verified by 12% PAGE, with RT-PCR positive products at 106 bp and negative specimens showing only primer dimers as expected.

The microfluidic assay for influenza A did not generate any false positives, including for those specimens shown to be RT-PCR positive for influenza B in bench top testing, resulting in a specificity of 100%. For specimens with greater than 10^5^ copies/ml viral loads, the sensitivity was 100%. Viral load was determined using absolute quantification by real time PCR (methods). The sensitivity decreased to 96% when specimens with viral loads down to 10^3^ copies/ml were included (**Table 1**). None of the specimens assayed had measured viral loads of less than 10^3^ copies/ml. Based on all specimens tested in the microfluidic assay (n = 146), the sensitivity and specificity were 96%*(CI 89%,99%)* and 100%*(CI 95%,100%)*, respectively (**Table 2**).

**Table 1:** Cumulative sensitivity and specificity of the microfluidic assay for influenza A for decreasing specimen viral loads.

No. of specimens (n) in each viral load “bin”	False negatives in each “bin”	Cumulative no. of specimens	Cumulative number of false negatives	Sensitivity	Specificity	Viral load (copies/ml)
1	0	1	0	100%	100%	10^10^
11	0	12	0	100%	100%	10^9^
29	0	41	0	100%	100%	10^8^
17	0	58	0	100%	100%	10^7^
8	0	66	0	100%	100%	10^6^
3	2	71	2	97%	100%	10^5^
2	1	74	3	96%	100%	10^4^
2	0	76	3	96%	100%	10^3^

**Table 2:** Summary statistics for the microfluidic assay.

Microfluidic Assay	*vs. Benchtop RT-PCR*			
(n = 146)	*positive*	*negative*		
*Positive*	**70**	**0**	100% *(94%,100%)*	**PPV**
*Negative*	**3**	**73**	96% *(88%,98%)*	**NPV**
	96% *(89%,99%)*	100% *(95%,100%)*		
	**Sensitivity**	**Specificity**		

*(95% CI)*.

**Figure 4:**
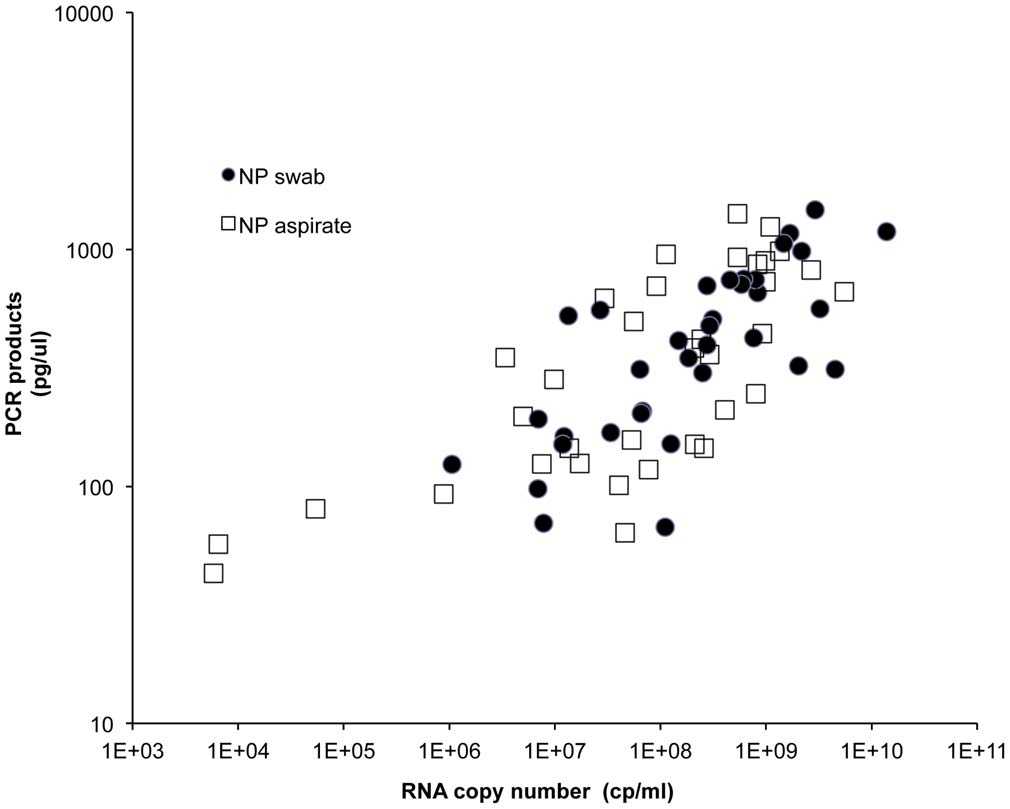
On-chip PCR product concentration vs. copy number showing NP swab and NP aspirate specimens.

As expected, there is significantly higher PCR product yield with increased RNA template input from higher viral loads (one-way ANOVA, p<0.001) (**Figure 4**). Two different specimen types (NPA and NPS) were tested in the microfluidic assay. Similar trends were found for both specimen types, with no observable difference between swab and aspirate samples (**Figure 4**).

### Other methods

To compare the performance of techniques in clinical use to the bench top RT-PCR performed in our laboratory, we selected additional specimens from our sample library that had been previously tested as part of routine clinical care using rapid immunoassays (Boston Medical Center) or DFA (Beth Israel Deaconess Medical Center). In total, n = 106 samples tested by DFA and n = 119 samples tested using rapid immunoassays were included in the testing and analysis. These sample sets are summarized in **Tables S2 and S3**. The DFA tests were 98%*(CI 91%,100%)* sensitive and 94%*(CI 86%,99%)* specific when compared to RT PCR run in our laboratory, with 1 false negative (NPV = 98%) and 2 potential false positives (PPV = 97%). The rapid immunoassays (Xpect Flu™ and BinaxNOW™) were 49%*(CI 38%,961%)* sensitive and 98%*(CI 90%,100%)* specific (n = 119), with 1 false positive (PPV = 97%) and 34 false negatives (NPV = 60%) (**Tables 3** **and** **4**).

**Table 3:** Summary statistics for DFA.

DFA	*vs. Benchtop RT-PCR*			
(n = 106)	*positive*	*negative*		
*Positive*	**58**	**2**	97% *(89%,99%)*	**PPV**
*negative*	**1**	**45**	98% *(89%,100%)*	**NPV**
	98% *(91%,100%)*	96% *(86%,99%)*		
	**Sensitivity**	**Specificity**		

*(95% CI).*

**Table 4:** Summary statistics for the rapid immunnoassays.

Rapid Immunoassays	*vs. Benchtop RT-PCR*			
(n = 119)	*positive*	*negative*		
*Positive*	**33**	**1**	97% *(85%,100%)*	**PPV**
*negative*	**34**	**51**	60% *(49%,70%)*	**NPV**
	49% *(38%,61%)*	98% *(90%,100%)*		
	**Sensitivity**	**Specificity**		

*(95% CI).*

## Discussion

Rapid, sensitive, and cost-effective detection of influenza virus from clinical specimens is critical for effective treatment and to limit the spread of infection. In this study, we miniaturized a nucleic acid amplification test into a single-use microfluidic chip to reduce the testing cost, decrease the turnaround time and move the PCR assay closer to true POC testing. We assessed the ability of the microfluidic chip to amplify influenza A virus RNA in clinical nasopharyngeal specimens.

146 specimens were chosen from the sample pool to test the microfluidic assay. Independently, two subsets of specimens that had been routinely tested using either a DFA (n = 106) or rapid immunoassay (n = 119) test as the standard of care were tested with the reference bench top RT-PCR assay. Compared to bench top RT-PCR as the gold standard, the microfluidic test performed with sensitivity and specificity comparable to the DFA assays that had been performed as standard of care, and with substantially greater clinical sensitivity (96% vs. 49%) than the rapid immunoassays that had been performed as standard of care. The 73 clinical specimens negative for influenza A by both bench top and microfluidic assays included 20 samples that were positive for influenza B by the bench top gold standard assay. Specimens with initial viral loads down to 10^3^ copies/ml were detected as positive in the microfluidic assay, although with reduced sensitivity (**Table 1**). Sensitivity was 100% for the specimens with influenza A copy numbers of greater than or equal to 10^5^ copies/ml and was 96% for specimens with copy numbers greater than or equal to 10^3^ copies/ml. Typical viral loads in the nasopharynx for patients recently infected with influenza A are rarely below 10^4^ copies/ml [41], [42], and this has been verified for several strains [43]. So, while there are several ways that the reported microfluidic assay could be improved to lower the limit of detection, in the case of influenza A infections, this improvement would be of limited benefit.

It is important to note that the low sensitivity of the rapid immunoassays was expected. Because in previous work by Pollock et al. [14], the DFA assay results were comparable to the performance of bench top RT-PCR, we closely examined our potential DFA false positive (2) and false negative (1) results. In the two DFA positive/bench top RT-PCR negative specimens, the patients who provided the specimens were clinically diagnosed with influenza based on compatible symptoms and signs, and each specimen was collected during the pH1N1 epidemic in Boston. One limitation of the bench top RT-PCR used in this study was that the assays were performed on the clinical specimens after they had undergone at least one freeze/thaw cycle, which may have led to some RNA degradation in the sample and thus slightly reduced the sensitivity of the gold standard RT-PCR. We hypothesize that this could potentially explain the two DFA-positive, bench top PCR-negative results. The DFA negative/bench top RT-PCR positive specimen was one which was already known to have low numbers of epithelial cells and was accordingly borderline inadequate for DFA testing [14]; a separate specimen from the same patient had tested positive for pH1N1 by RT-PCR testing done at the Massachusetts State Laboratory, confirming our own bench top RT-PCR results.

We plotted the input viral load as determined by the bench top quantitative PCR against the amplicon output concentration from the microfluidic assay measured using capillary electrophoresis (**Figure 4**). Since these were endpoint reads, we did not expect a strong linear relationship between input copy number and endpoint concentration (linear regression R^2^ = 0.174 for NPA specimens and R^2^ = 0.253 for NPS specimens), and we did not see one.

All of the microfluidic assays were run with the same primer, enzyme and blocking agent starting concentrations. These concentrations were chosen based on a series of preliminary optimization experiments that are described in the data (**Figures S1, S2, S3, S4**). The only variable in the reaction was the input sample, which varied in several ways. First, there was variation in the viral load between specimens. There were also differences in sample type (NPA vs. NPS) and in the method of storage before microfluidic testing. The samples collected at BMC were aliquotted and stored in VTM, while almost all of the samples collected at BIDMC were not aliquotted and were stored in PBS (a minority of specimens were stored in VTM). The aliquotting allowed for minimal freeze thaws before testing the samples in the reference bench top RT-PCR assay and the microfluidic chip assay. Most of the samples went through one freeze/thaw cycle before microfluidic testing and the bench top RT-PCR. In addition, all samples were spun once before introduction into the microfluidic chip. The BMC samples were spun before aliquots were frozen, and the BIDMC samples were spun after aliquots were thawed fore testing in the microfluidic assay. This “preprocessing” step was performed to minimize the chance of clogging the nucleic acid extraction part of the chip with cell debris, which was seen in a small number (about 10%) of the preliminary experiments due to the very small pore size of the SPE columns used in these chips. Recent reformulations of the SPE in our lab have resulted in larger average pore sizes that have eliminated the need for this initial spin down step. It is possible, however that the spin step also acted to remove PCR inhibitors, resulting in better results than had the samples been left completely untreated.

The PCR channel design used in the microfluidic assay was optimized previously to achieve repeatable and predictable temperature profiles in the chip [44]. The serpentine PCR channel flows the sample through 30 cycles of PCR. The cycle number is fixed for any given chip, unlike in bench top PCR schemes. Increasing the cycle number (by redesigning the chip) and/or reducing the flow rate could further improve the PCR yield, but testing time would also increase. So, for any given assay, the chip design and the PCR reaction itself must be optimized simultaneously. Here, a validated protocol obtained from the CDC was adapted for use in the microfluidic chip. Only the influenza A primer set from the CDC protocol was used in the microfluidic assay. In place of the other reagents, we used reagents and buffers from the Qiagen One-Step RT-PCR Kit, which is specially designed to enable reactions over a wide range of Mg^2+^ concentrations through a balanced combination of KCl and (NH_4_)_2_SO_4_ [45]. The standard Qiagen assay was further optimized by adding twice the amount of enzyme and more BSA as a blocking agent to improve amplification in a high surface to volume ratio environment (**Figures S2, S3, S4**). It has been observed by others that enzyme can bind non-specifically to the walls and become inactive in high surface to volume ratio reaction chambers [46].

Key advantages of an integrated microfluidic chip are rapid turnaround time, low cost, potential for portability (and thus true point-of-care use) and reduced possibility of sample-to-sample contamination. While much faster than most clinical laboratory turn around times for molecular assays (which usually run more than 4 hours), 3 hours is still too long for a true POC test. There are several avenues available to improve the assay and reduce the testing time. To meet the demands of a POC setting (e.g. clinician's office), the end-to-end time for the assay must be short (less than one hour) and the hands-on time very short. The hands-on time must include as few steps as possible, and the steps should be easily performed by a minimally trained user. This end-to-end time can be markedly reduced in our system through further optimization. The loading and washing times could be reduced by optimizing SPE compositions in the chip to allow for faster fluid flow (larger pore sizes) and including multiple shorter columns in parallel instead of one long column. These improvements are expected shorten the loading and washing of the SPE to about 20 min. To reduce the PCR time, continuous flow PCR with real time reading at each turn of the serpentine channel could further reduce the cycling time by allowing the reader to see a positive result before all 30 cycles of PCR were complete. With these two modifications, the total turn-around-time could be reduced to less than 1 hour making the assay suitable for true POC applications.

As in any proof of concept design, there are limitations in the current embodiment. First, none of the reagents were stored on chip and were added as required while running the assay. Lyophilized reagents for RT and PCR are commercially available and will be required for a self-contained system. Further, if quantitative results are sought, the endpoint read is not a good method, due to the saturation of PCR at high cycle numbers. Quantitative results may not be useful in the case of influenza, but are absolutely critical for other RNA virus diagnostic tests, including those for HIV and HCV. Continuing efforts include adding real time detection through the addition of fluorescence imaging at several locations on the chip. Further, multiplex PCR or spatial multiplexing, although not demonstrated on this device, would greatly enhance the power of this technique and would be imperative for an optimal influenza assay (i.e. the ability to detect and distinguish influenza A and B).

Although this microfluidic chip shares some similarities with other devices [47], it has several unique characteristics. This chip is capable of analyzing clinical specimens directly with minimal sample preparation. Here, we did spin down the initial sample once before applying it to the microfluidic chip. This step was performed to avoid clogging of the SPE columns by cell debris that might be present in the sample, and it can be eliminated by using SPE columns with larger average pore sizes as described above. The chip has a single layer design and is made from a thermoplastic material suitable for high throughput fabrication using injection molding. Last, the fluidic and thermal controls are very simple. Continuous-flow PCR eliminates the need to thermocycle the entire chip. The flow is almost entirely regulated by differential flow resistance between the functional chambers. We demonstrated the feasibility of very simple control methods using one pump, two heaters and 3 thermocouples. These items would be packaged in a “reader” for an eventual stand-alone test.

In summary, a newly developed single use microfluidic assay was demonstrated to amplify influenza A RNA in clinical specimens with high sensitivity and specificity, with performance essentially equivalent to standard bench-top RT-PCR. The microfluidic assay is simple to fabricate and to run, since the microfluidic device is a single layer with a cover, and the assay steps follow in a linear progression without active valving on chip. The sample introduction, static heating and pressure driven flow have been automated with our collaborators for a very similar bacterial detection assay [48] and these improvements could be easily applied to the system demonstrated here. The parallel development of a very simple, low cost reader with the form factor of a clinical digital thermometer would meet the requirements for bedside use. We see these results as a major step toward a true POC molecular assay for influenza A, and this proof of concept demonstration as a basis for the molecular detection and diagnosis of a number of other infections.

## Materials and Methods

### Specimens and specimen characterization

A total of 626 NPA and NPS samples were collected during the 2008–2010 influenza seasons including the 2009 pandemic period. The specimens were collected from adult and pediatric patients at two hospitals in Boston, Massachusetts, USA; 383 from adult and pediatric patients in the emergency room at Boston Medical Center (BMC) and 243 from a combination of inpatients and outpatients (almost all adults) from Beth Israel Deaconess Medical Center (BIDMC). Each study was reviewed and approved by the site's institutional review board. The specimens collected at BMC were collected prospectively and frozen; the specimens from BIDMC were frozen discarded specimens that had been collected and tested during routine clinical care. At BMC, informed consent was obtained from all participants. All patients were consented in writing and had the ability to withdraw from the study at any time. Pediatric patients were consented in writing by their parent or legal guardian in addition to being asked for verbal assent if they were able. At both BMC and BIDMC, specimens were collected from patients presenting with one or more symptoms consistent with influenza, including fever, cough, sore throat, myalgia, nasal congestion or rhinorrhea (runny nose), headache, malaise, or diarrhea. Subjects had a distribution of ages ranging from 12 months to 70 years (as noted, specimens obtained from BIDMC were almost exclusively from adults) and included both genders. No subject was excluded based on race.

At BMC, NPA samples were taken from consented subjects following standard techniques [49], [50]. Briefly, the person obtaining the sample measured the distance from the patient's nostril to the nasopharynx and held the tube at that location. A 0.6 mm×19 mm×305 mm flexible sterile suction catheter was placed through the nostril to the posterior nasopharynx. One to 2 ml of sterile saline was instilled through the catheter and the aspirate was immediately collected with gentle suction while simultaneously removing the catheter. The NPA was collected into a sterile container of 1.5 ml of viral transport medium (VTM) (BD, Franklin Lakes, NJ). Specimens were stored at BMC for no longer than 72 hours at 4°C before deidentification, transfer and storage at the Klapperich Laboratory. Specimens received at the Klapperich Laboratory were immediately centrifuged at 5000 rpm for 5 min and stored as 0.5 ml aliquots at −80°C. They were not spun again before testing. A subset of the samples was tested using rapid immunoassays as part of the standard of care, by request of the treating physician. When a rapid test was performed, the remainder of the sample was sent to the Klapperich Laboratory for further testing.

Both NPA and NPS samples were taken at BIDMC, though the vast majority of the specimens were swab (NPS) samples. In all cases, specimens were taken from patients in the course of routine clinical care, for testing ordered by the patient's clinician. NPS specimens were taken using two Copan flocked swabs (COPAN, Murrieta, CA). The first swab was inserted flat and pushed forward with gentle downward pressure on the lower nasal floor to the posterior wall of the nasopharynx, where it was rotated for a few seconds to collect cellular material. The swab was withdrawn and placed into sterile 1X PBS. The collection procedure was repeated using the second flocked swab in the other nostril; the second swab was placed into M4RT (Remel, Lenexa, KS) media for viral culture. The two swabs were then submitted on ice to the BIDMC microbiology laboratory. After routine direct fluorescent antigen (DFA, performed on the PBS tube in all cases [14]) and culture (performed on the M4 tube in a subset of cases) testing, tested/resulted specimens (approximately 1.0 mL) were stored at −80°C. In a minority of patients, NPA specimens were collected instead of NPS specimens and submitted on ice to the BIDMC clinical microbiology laboratory for testing, after which they were frozen at −80°C. The frozen (discarded) NPS and NPA specimens were later deidentified and sent to the Klapperich Laboratory where they continued to be stored at −80°C. Before testing, all specimens were routinely quick thawed from −80°C in a 37°C water bath. Then the specimens were spun at 13,000 rpm for 10 min at 4°C to remove human cell debris and mucous, and the supernatant was used for all downstream testing.

### Microfluidic Chip Fabrication

The microfluidic chips were fabricated in thermoplastic Zeonex 690R (Zeon Chemicals, Louisville, KY), by hot-embossing a blank 0.7 mm thick Zeonex plaque using an epoxy master mold and then thermally sealing the imprinted channel with a 0.7 mm blank Zeonex cover slip [51]. Three open ports in the chip were fitted with nanoports (Upchurch Scientific, Oak Harbor, WA) using epoxy glue to facilitate loading the specimen, adding the reagents and collecting the RT-PCR products. The entire chip is 70 mm in length, 25 mm in width and 1.4 mm in height. The integrated chip consists of three functional chambers, a solid phase extraction (SPE) column for RNA extraction, an RT chamber, and the PCR channel. The sample preparation channel was fabricated following methods described in earlier work [52], [53]. All reagents for SPE column fabrication were purchased from Sigma (St. Louis, MO) unless otherwise noted. Before fabrication of the SPE column, the channel was cleaned with 50 µl RNAse Away, (Molecular BioProducts, San Diego, CA), and then rinsed with 100 µl nuclease free water (Promega, Madison, WI). Briefly, silica microspheres (0.7 µm, cat# 24324-15, Polysciences Inc.) were prepared by centrifugation for 5 min at 6600 rpm, aspirating the suspension solution, and drying on a heat block overnight to remove excess water. The inner channel surface was grafted with 97% v/v methyl methacrylate and 3% v/v benzophenone by 10 min of UV-irradiation at 265 nm in an Ultraviolet Crosslinker (CL-1000, UPV Inc., Upland, CA). This step facilitated covalent attachment between the SPE column and the inert plastic channel surface. The SPE column itself was made with a mixture of 16% v/v ethylene dimethacrylate, 24% butyl methacrylate, 42% 1-dodecanol, 18% cyclohexanol, and 1% photoinitiator 2-dimethylamino-4-(methyl-phenylamino)-phenol. Silica microspheres were added into the monolith solution in a 1∶1 ratio (v/v). The monolith was then cross-linked by UV irradiation for 5 min as above through both sides of the chip, achieved by flipping the chip over half way through irradiation. Before use, chips were cleaned with 500 µL of 100% methanol to rinse away excess porogen and to create the open pore structure of the SPE.

### The Microfluidic Assay

The first chamber of the chip is an SPE column for sample preparation; it is integrated directly with the RT and PCR steps. Sample preparation followed the same strategy as previously reported [54]. Briefly, before introducing the sample, the channel was conditioned with 300 µL of buffer containing 1.5 M guanidine thiocyanate (GuSCN) (Sigma, St. Louis, MO), 50% 2-Propanol (Sigma), and 1X RNASecure (Applied Biosystems, Foster City, CA). Next, 100 µl of the NPA or NPS specimen was mixed with 300 µl lysis buffer (2 M GuSCN, 66.7% 2-propanol, 1x RNASecure, 6 µg carrier RNA (Qiagen, Valencia, CA)). This mixture was run though the channel and collected from the first outlet port. During this step, nucleic acids released from the lysed influenza particles in the specimen bind to the SPE column. The SPE channel was then cleaned sequentially with 50 µl of 70% ethanol followed by 50 µl of 100% ethanol, which were also collected at the first outlet (to waste). The residual ethanol was removed by passing 500 µL of dry air through the channel. Finally, the extracted RNA was eluted in 13–15 µl of nuclease free water. The flow rates for all steps were between 0.8–1 ml/hr. All fluids were pushed using a programmable syringe pump (PHD2000, Harvard Apparatus, MA).

The extracted RNA was amplified by RT-PCR on-chip using reagents from the One Step RT-PCR kit (Qiagen). The CDC influenza A primers were used (see below), targeting the highly conserved M1 gene and generating a 106 bp amplicon [39]. Each 50 µl RT-PCR reaction included 13.5 µl RNA, 4 µl one step RT-PCR enzyme mix, and the following reagents with the given final concentrations: 1X Q solution, 1X one step buffer, 1 mM additional MgCl_2_, 1 mM forward primer 5′-GAC CRA TCC TGT CAC CTC TGA C-3′, 1 mM reverse primer 5′-AGG GCA TTY TGG ACA AAK CGT CTA-3′, 400 µM dNTP, 0.015% w/v BSA, and 0.75% w/v PEG8000. The PCR products were analyzed using a 2100 Bioanalyzer and sequenced using the PCR primers. Influenza A/PR/8/34 cultured in Madin-Darby canine kidney (MDCK) cells was used as the chip positive control and nuclease free water was the chip negative control.

After the extracted RNA was eluted and collected in the well at the end of the SPE channel, the RT-PCR reagents were loaded, and the solution was pushed into the RT chamber. The RT chamber was maintained at 50°C for 30 min, followed by 95°C for 15 min to activate the PCR enzyme according to the manufacturer's protocol. Next the reaction flowed through the continuous-flow PCR channel for amplification. A denaturation temperature of 95°C and annealing temperature at 60°C were applied through the thin film heaters. The flow rate was 0.6 µl/min, resulting in a 40 sec cycle time. The reaction products were collected in the second outlet port 30 cycles (20 min).

### Bench Top Quantitative PCR

For the off-chip control reactions, RNA was extracted from 140 µl of each specimen using the QIAamp Viral RNA Mini Kit (Qiagen) as per the manufacturer's protocol. First strand cDNA synthesis (RT) and amplification (PCR) steps were combined by using the SuperScript III Platinum® One-Step qRT-PCR Kit w/ROX (Invitrogen, Carlsbad, CA). The pan A Universal primers and probes used were (Biosearch Technologies, Novato, CA) influenza A forward primer: 5′ GAC CRA TCC TGT CAC CTC TGA C 3′, reverse primer: 5′ AGG GCA TTY TGG ACA AAK CGT CTA 3′, and probe: 5′ TGC AGT CCT CGC TCA CTG GGC ACG 3′. For influenza B, the primers were forward primer: 5′ TCC TCA AYT CAC TCT TCG AGC G 3′, reverse primer: 5′ CGG TGC TCT TGA CCA AAT TGG 3′, and probe: 5′ CCA ATT CGA GCA GCT GAA ACT GCG GTG 3′ [39]. All reactions were performed in a 7300 Real Time PCR system (Applied Biosystems, Foster City, CA) under the following conditions: 30 min at 50°C for RT, 15 min at 95°C for polymerase activation, followed by 45 cycles of 15 sec at 95°C and 60 s at 55°C for amplification. The primers, reagents and amplification scheme are as described in the CDC protocol for real time RT-PCR for influenza A and B (H1N1) [39].

A standard curve was established for each qRT-PCR using plasmids containing a single copy of the influenza A M1 sequence targeted by the PCR primers (SeqWright, Houston, TX). The plasmid was generated by cloning the M1 PCR product with the pCR 2.1 TOPO-TA cloning kit (Invitrogen) and quantified with PicoGreen to prepare plasmid dilutions of 10^7^ to 10^1^copies/µl. The viral cDNA copy number generated from a given patient specimen was determined by interpolation from a standard curve of cycle threshold (C_T_) values based on the known input concentrations of plasmid DNA.

### Microfluidic chip product validation

Results available from the clinical laboratories at each site varied (DFA results were available for all specimens from BIDMC, and rapid assay results were available for a subset of cases from BMC specimens). In order to establish a “gold standard” method across all of the samples, we re-tested all of the samples using standard bench top quantitative RT-PCR for both influenza A and influenza B. The results of this quantitative PCR were used to calculate the statistical performance of the microfluidic assay.

For the microfluidic assays, positive and negative specimens were tested in a random order, and each specimen was tested on a new chip to avoid cross contamination. The 146 samples selected for testing with the microfluidic assay were not chosen with regard to viral load or any other parameter. Previously unthawed aliquots were preferentially used to reduce potential viral degradation in the sample. The concentration and product size of microfluidic assay PCR products from all specimens was analyzed by gel capillary electrophoresis on a 2100 Bioanalyzer (Agilent, Santa Clara, CA) with the High Sensitivity DNA Kit (Agilent). The correct product size was also confirmed for all positive samples by 12% PAGE. Every 10^th^ negative sample from each week was verified as negative by 12% PAGE.

Every 10^th^ positive sample was sequenced (SeqWright, Houston, TX or Genewiz, South Plainfield, NJ) to verify the product sequence as the correct fragment of the M1 gene of influenza A. The PCR products (10 µl) were cleaned up with ExoSAP-It® (USB Scientific, CA) and then sequenced using PCR primers. Sequences from the chip amplicons were analyzed by standard methods. Briefly, all GenBank influenza type A M1 gene sequences from 2008–2010, from humans in North America (n = 739) were retrieved from the Influenza Virus Resource database at the National Center for Biotechnology Information (NCBI) [40]. The sequences were aligned using ClustalX [55] using the M1 sequence of influenza A/PR/8/34 (accession number NC_002016) as the reference.

### Statistical Analysis

A total of 73 influenza A positive patient specimens and 73 influenza A negative patient specimens were selected from the banked samples and were tested using the microfluidic assay. We targeted demonstration of 95% sensitivity. In order to have the statistical power to demonstrate sensitivity 95% or better, with a prevalence of 50% (input sample pool was adjusted) with a confidence interval of 0.05, a sample size of 146 was required. The 73 negative samples included some samples that were negative for influenza A, but positive for influenza B by the bench top RT-PCR assay. The sensitivity, specificity, positive predictive value (PPV), and negative predictive value (NPV) were determined using standard methods [56] and are reported as comparisons to standard bench top qRT-PCR, which is emerging as the gold standard test method [42], [57]. One-way ANOVA (95% CI) was performed to determine the significance of relationships between viral load and sample type (NPA or NPS) on the microfluidic assay PCR product yield. Microsoft Excel and GraphPad Prism (v5, La Jolla, CA) were used for the analyses.

### Rapid immunoassays and DFA

The Xpect™ Flu A & B kit (Remel, Lenexa, KS) and the BinaxNOW™ Influenza A & B kit (Inverness Medical, Princeton, NJ) were used as per the manufacturer's protocols at the BMC emergency departments. These tests were ordered at the discretion of the attending clinician. The rapid immunoassay tests were performed using a small amount of the same NPA collected for this study. DFA assays were conducted at BIDMC as previously described [14].

## Supporting Information

Figure S1 **Microfluidic assay performance using cultured influenza A virus and standard Qiagen OneStep Kit protocol.**
(TIF)

Figure S2 **Microfluidic assay performance using cultured influenza A as a function of increasing BSA concentrations.** The baseline assay contains no additional BSA.
(TIF)

Figure S3 **Microfluidic assay performance as a function of increasing MgCl_2_ concentration.** The baseline assay has 2.5 mM of MgCl_2_.
(TIF)

Figure S4 **Microfluidic assay performance as a function of increasing enzyme concentration.** The baseline assay contains 2 µl of the enzyme master mix. The manufacturer does not give the concentration in IU.
(TIF)

Table S1 **Summary of the patient specimens tested in the microfluidic assay and bench top RT-PCR.**
(XLS)

Table S2 **Summary of the patient specimens tested by DFA and bench top RT-PCR.**
(XLS)

Table S3 **Summary of the patient specimens tested by rapid immunoassay and bench top RT-PCR.**
(XLS)
