# Supplementary figures and images for: Microfluidic Chip for Molecular Amplification of Influenza A RNA in Human Respiratory Specimens
Source: PLoS One. 2012 Mar 22;7(3):e33176. doi: 10.1371/journal.pone.0033176 (PMC3310856; doi:10.1371/journal.pone.0033176)

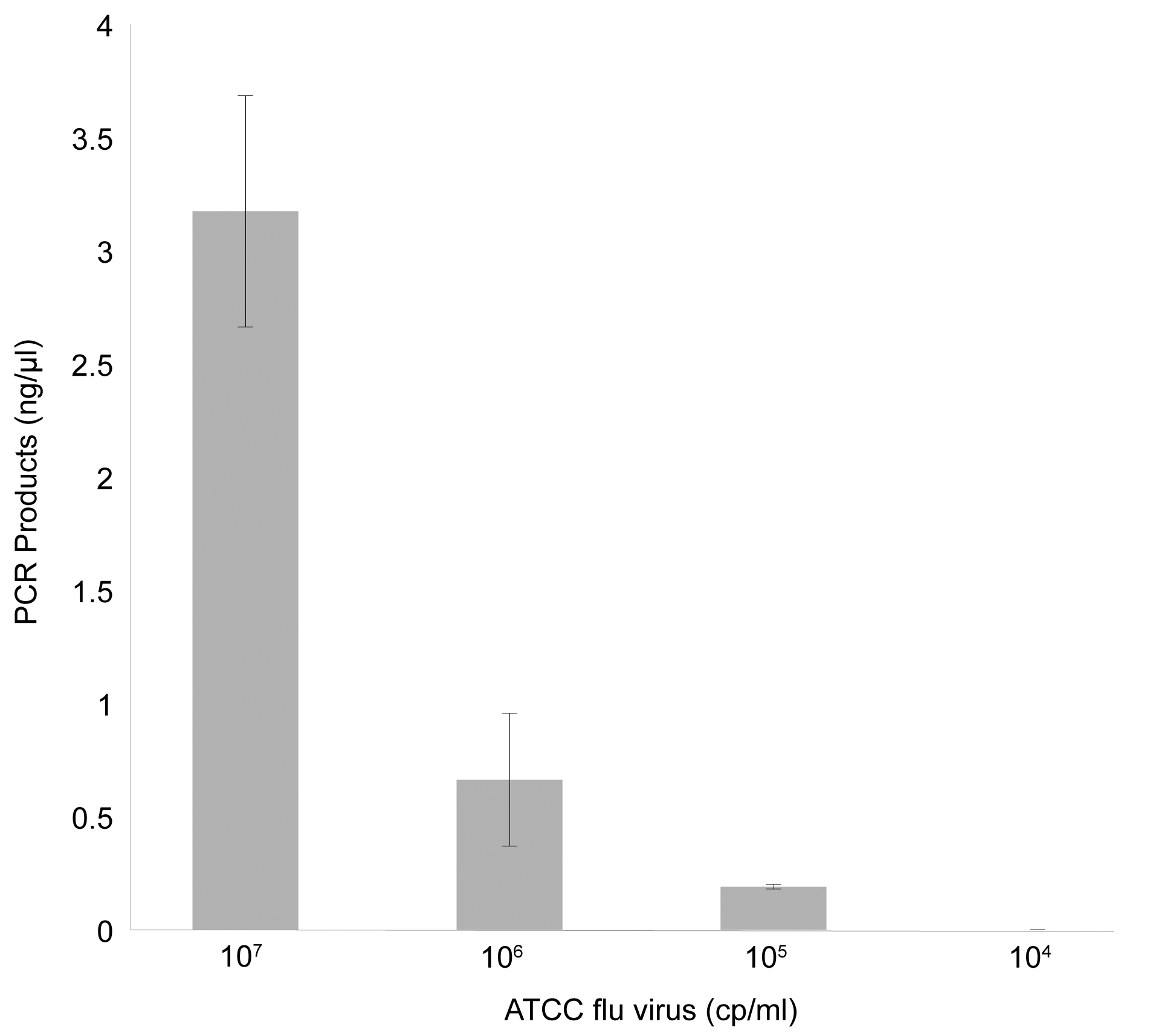

Supplement: Figure S1 — Microfluidic assay performance using cultured influenza A virus and standard Qiagen OneStep Kit protocol. (TIF) [file pone.0033176.s001.tif]

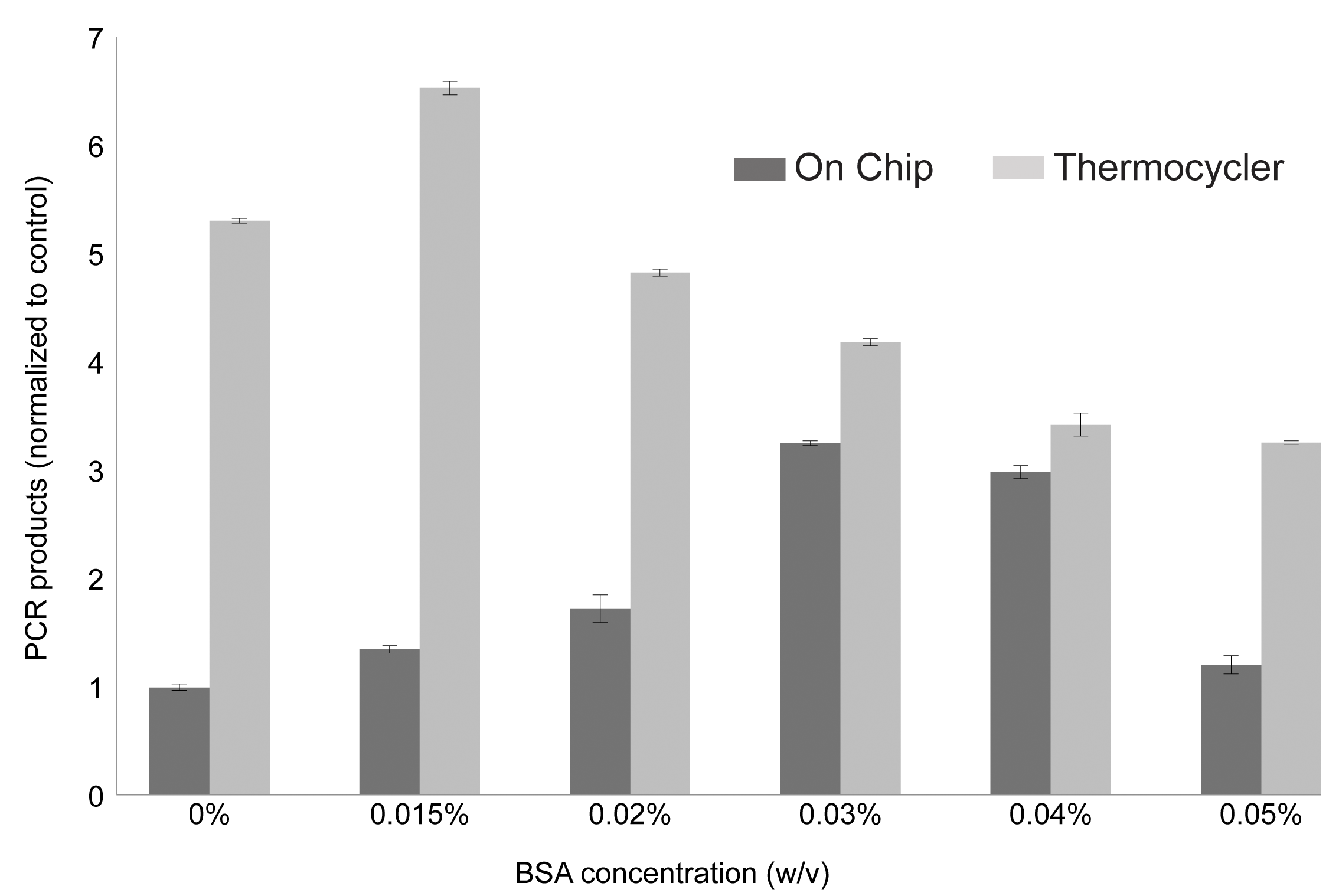

Supplement: Figure S2 — Microfluidic assay performance using cultured influenza A as a function of increasing BSA concentrations. The baseline assay contains no additional BSA. (TIF) [file pone.0033176.s002.tif]

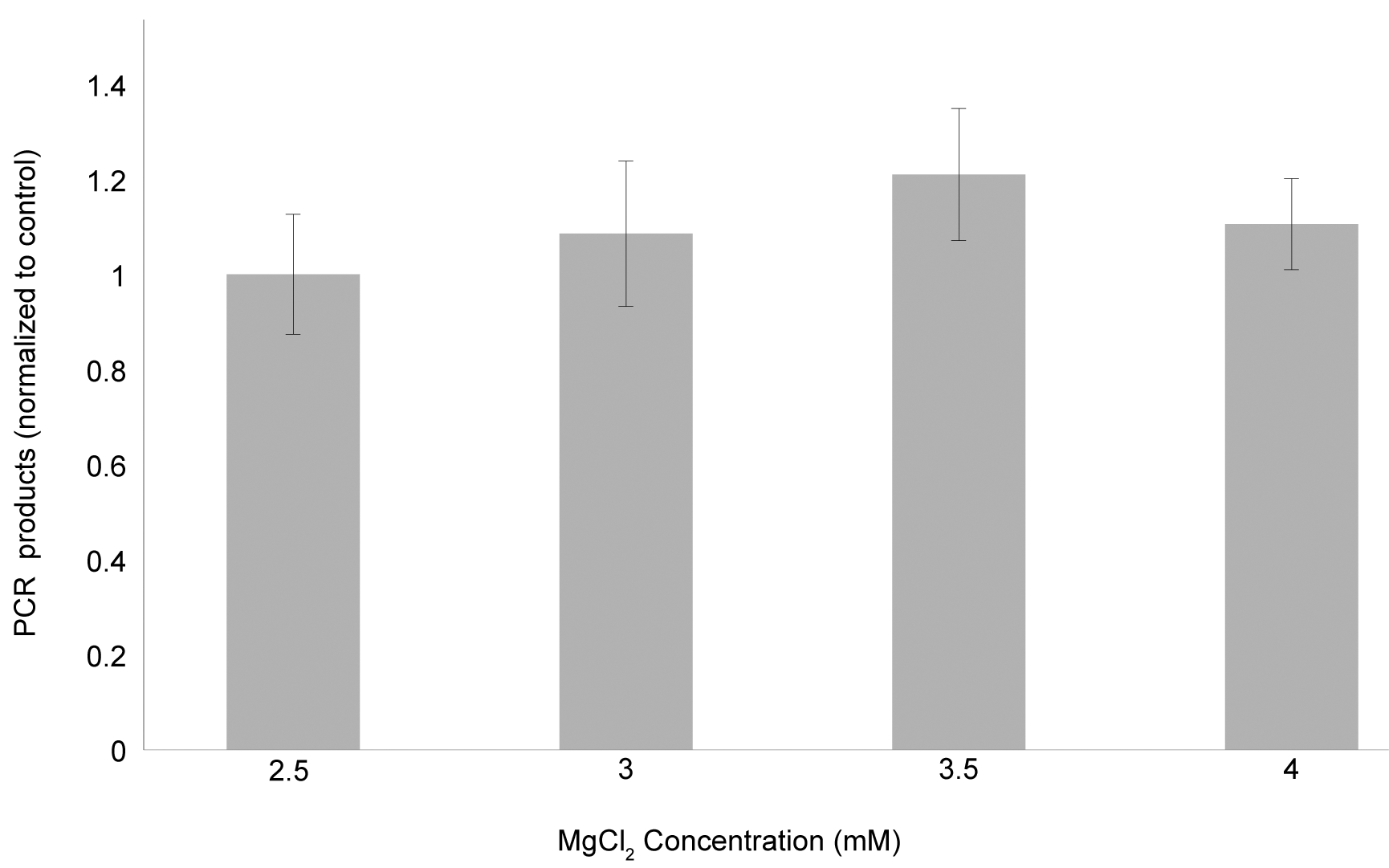

Supplement: Figure S3 — Microfluidic assay performance as a function of increasing MgCl2 concentration. The baseline assay has 2.5 mM of MgCl2. (TIF) [file pone.0033176.s003.tif]

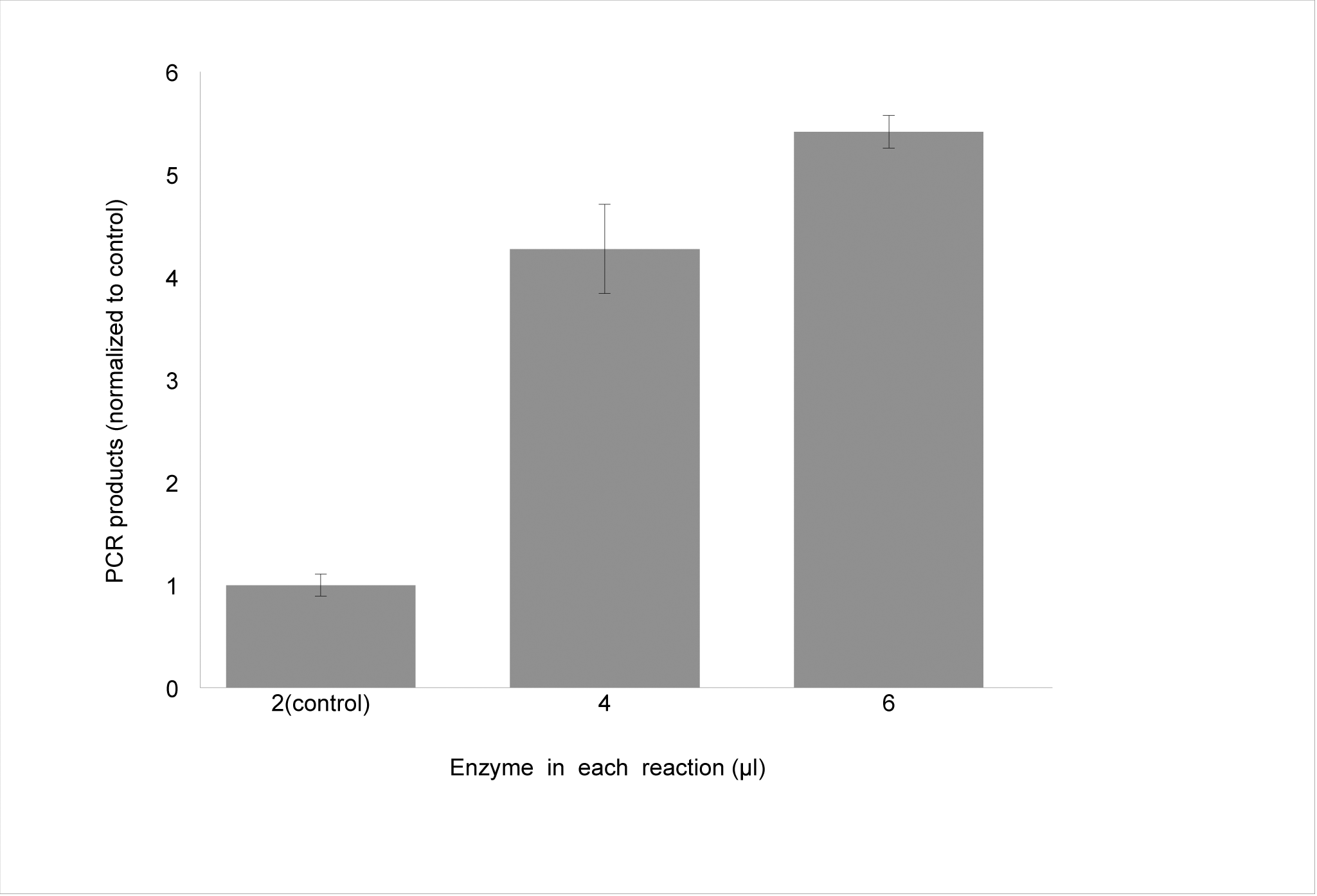

Supplement: Figure S4 — Microfluidic assay performance as a function of increasing enzyme concentration. The baseline assay contains 2 µl of the enzyme master mix. The manufacturer does not give the concentration in IU. (TIF) [file pone.0033176.s004.tif]
